# Targeting Sentinel Proteins and Extrasynaptic Glutamate Receptors: a Therapeutic Strategy for Preventing the Effects Elicited by Perinatal Asphyxia?

**DOI:** 10.1007/s12640-017-9795-9

**Published:** 2017-08-26

**Authors:** Mario Herrera-Marschitz, Ronald Perez-Lobos, Carolyne Lespay-Rebolledo, Andrea Tapia-Bustos, Emmanuel Casanova-Ortiz, Paola Morales, Jose-Luis Valdes, Diego Bustamante, Bruce K. Cassels

**Affiliations:** 1 Programme of Molecular & Clinical Pharmacology, ICBM, Faculty of Medicine, University of Chile, Av. Independencia, PO Box 8389100, 1027 Santiago, Chile; 2 Escuela de Tecnologia Medica, Facultad de Medicina, Universidad Andres Bello, PO Box 8370146, Santiago, Chile; 3 Faculty of Sciences, University of Chile, Santiago, Chile; 4 Department of Neuroscience, Faculty of Medicine, University of Chile, Santiago, Chile

**Keywords:** Neonatal hypoxia, Hypoxic ischaemic encephalopathy (HIE), Leukomalacia, Basal ganglia, MAP-2, GFAP, TUNEL, nNOS, Delayed cell death, Organotypic cultures, Niacinamide, Memantine, Rat

## Abstract

Perinatal asphyxia (PA) is a relevant cause of death at the time of labour, and when survival is stabilised, associated with short- and long-term developmental disabilities, requiring inordinate care by health systems and families. Its prevalence is high (1 to 10/1000 live births) worldwide. At present, there are few therapeutic options, apart from hypothermia, that regrettably provides only limited protection if applied shortly after the insult.

PA implies a primary and a secondary insult. The primary insult relates to the lack of oxygen, and the secondary one to the oxidative stress triggered by re-oxygenation, formation of reactive oxygen (ROS) and reactive nitrogen (RNS) species, and overactivation of glutamate receptors and mitochondrial deficiencies. PA induces overactivation of a number of sentinel proteins, including hypoxia-induced factor-1α (HIF-1α) and the genome-protecting poly(ADP-ribose) polymerase-1 (PARP-1). Upon activation, PARP-1 consumes high amounts of ATP at a time when this metabolite is scarce, worsening in turn the energy crisis elicited by asphyxia. The energy crisis also impairs ATP-dependent transport, including glutamate re-uptake by astroglia. Nicotinamide, a PARP-1 inhibitor, protects against the metabolic cascade elicited by the primary stage, avoiding NAD^+^ exhaustion and the energetic crisis. Upon re-oxygenation, however, oxidative stress leads to nuclear translocation of the NF-κB subunit p65, overexpression of the pro-inflammatory cytokines IL-1β and TNF-α, and glutamate-excitotoxicity, due to impairment of glial-glutamate transport, extracellular glutamate overflow, and overactivation of NMDA receptors, mainly of the extrasynaptic type. This leads to calcium influx, mitochondrial impairment, and inactivation of antioxidant enzymes, increasing further the activity of pro-oxidant enzymes, thereby making the surviving neonate vulnerable to recurrent metabolic insults whenever oxidative stress is involved. Here, we discuss evidence showing that (i) inhibition of PARP-1 overactivation by nicotinamide and (ii) inhibition of extrasynaptic NMDA receptor overactivation by memantine can prevent the short- and long-term consequences of PA. These hypotheses have been evaluated in a rat preclinical model of PA, aiming to identify the metabolic cascades responsible for the long-term consequences induced by the insult, also assessing postnatal vulnerability to recurrent oxidative insults. Thus, we present and discuss evidence demonstrating that PA induces long-term changes in metabolic pathways related to energy and oxidative stress, priming vulnerability of cells with both the neuronal and the glial phenotype. The effects induced by PA are region dependent, the substantia nigra being particularly prone to cell death. The issue of short- and long-term consequences of PA provides a framework for addressing a fundamental issue referred to plasticity of the CNS, since the perinatal insult triggers a domino-like sequence of events making the developing individual vulnerable to recurrent adverse conditions, decreasing his/her coping repertoire because of a relevant insult occurring at birth.

## The Problem

Pregnancy culminates at the time when labour begins, implying a complex interchange of molecules generated by uterine and extrauterine tissue, leading to increased myometrial contractility, cervical dilatation, decidual/membrane activation, and rupture of chorioamniotic membranes (Romero et al. 2006). The switch from a quiescent to a contractile myometrium is accompanied by a shift from anti-inflammatory to pro-inflammatory signalling chemokines and cytokines, as well as contraction-associated proteins, warranting a successful delivery (Romero et al. 2014). Delivery, however, can be a risky episode, whenever the onset of pulmonary respiration is delayed or interrupted, leading to perinatal asphyxia (PA) if oxygenation is not promptly established or re-established.

PA is a relevant cause of death at the time of labour, associated with long-term consequences when re-oxygenation is established (Odd et al. 2009). Despite important advances in perinatal care (Kurinczuk et al. 2010; Basovich 2010), PA remains a severe condition, with high prevalence (1 to 10/1000 live births) worldwide, also associated with long-lasting neuropsychiatric dysfunctions when children reach critical developmental stages (see Douglas-Escobar and Weiss 2015).

PA implies a deregulation of gas exchange resulting in hypoxemia, hypercapnia, and metabolic acidosis of vital organs, including the brain (Low 2004). The interruption of oxygen supply causes energy failure, triggering a biochemical cascade leading to cell dysfunction and ultimately to cell death, particularly affecting neurocircuitries of the basal ganglia and hippocampus (Klawitter et al. 2007; Morales et al. 2008; Neira-Peña et al. 2015). The long-term effects observed after PA also imply metabolic and neuronal network alterations, impairing the ability of the CNS to cope with stressors occurring during life (see Marriott et al. 2017).

Hypoxia leads to generation of reactive oxygen (ROS) and reactive nitrogen (RNS) species, inhibiting prolyl-hydroxylases that under normoxia metabolise the oxygen sensor *hypoxia-inducible factor-1alpha* (HIF-1α). This is then poly-ubiquinated by von Hippel-Lindau tumour-suppressing factor (pVHL) and eliminated by the proteasome (Wang et al. 1995). Following the interruption of oxygen viability, HIF-1α accumulates and translocates to the nucleus, stimulating the expression of multiple genes associated with cell metabolism and mitochondrial function, down-regulating the citric acid cycle and enhancing anaerobic glycolysis, thus allowing the cells to cope with the low oxygen tension (Ke and Costa 2006; Vangeison et al. 2008). HIF-1α translocation stimulates pro-apoptotic genes, including the Bcl-2 family members Nix, Noxa, Bnip3, and apoptosis-inducing factor (AIF) (Bruick 2000; Sowter et al. 2001) but also the expression of sentinel proteins, such as poly(ADP-ribose) polymerase-1 (PARP-1). PARP-1 signalling occurs via the attachment of ADP-ribose chains to nuclear proteins recognised by DNA-repairing enzymes, such as DNA ligase III. The generation of ADP-ribose monomers requires, however, NAD^+^, which is why PARP-1 overactivation further depletes NAD^+^ stores, resulting in progressive ATP depletion (Berger 1985; Hong et al. 2004). Furthermore, there is tight crosstalk between PARP-1 and HIF-1α (Martin-Oliva et al. 2006). Under hypoxic conditions and/or oxidative stress, PARP-1 modulates HIF-1α activity (Martinez-Romero et al. 2009). In turn, HIF-1α requires PARP-1 activation for exerting its transcriptional activity (Pan et al. 2013), while PARP-1 activity protects the HIF-2α isoform against pVHL-mediated destabilization (Gonzalez-Flores et al. 2014).

Hypoxia implies a generalised impairment of Na^+^/K^+^-ATPase-dependent transport, including neurotransmitter re-uptake. A particular case is that of glutamate, which is largely synthesised by the astroglia-neuronal glutamine shuttle. It is not yet clear how ATPase modulates glutamate transport. However, arachidonic acid inhibits several sodium-coupled amino acid transporters, including that of glutamate, by a mechanism requiring Na^+^/K^+^-ATPase (Danbolt 2001). Furthermore, there is evidence showing that extracellular glutamate levels are buffered by ATP-dependent transport, to be taken up by glial and neuronal cells for metabolic degradation or re-cycling (Herrera-Marschitz et al. 1996). ATP deficit decreases glutamate uptake, resulting in increased extracellular glutamate levels. Free radicals can also affect the members of the Na^+^/Cl^−^-dependent transporter family, although the role of oxidative modulation of glutamate uptake under normal conditions is not yet known, and even less under hypoxia (Danbolt 2001). It has been shown, however, that glutamate transporters possess a sulfhydryl-based regulatory mechanism, which makes glutamate transporters sensitive to redox agents, resulting in increased or decreased transport (Trotti et al. 1997). It is hypothesised here that under sustained hypoxia the half-life of extracellular glutamate is prolonged. This might provide an extreme homeostatic response for widespread neuronal depolarization removing the organism from a catastrophic condition by extracellular glutamate binding to any available glutamate receptor, mainly of the extrasynaptic subtype. The NMDARs are heterotetramers composed by two NR1 (obligatory) and two NR2/3 subunits (Jacobucci and Popescu 2017), whose gating and ligand-binding properties depend on the NR2A/C subunit (Glasow et al. 2015). The NR2B-containing NMDARs are extrasynaptic in a significant proportion (Papouin et al. 2012). At birth, extrasynaptic NR2B-containing receptors prevail over the NR2A-containing NMDAR subtype. The NR2A-containing subtype is the predominant intrasynaptic mature NMDAR, associated with long-term plasticity (see Petralia 2012; Vizi et al. 2013). The NR2B subtype is associated with excitotoxic cascades and cell death, via Ca^2+^ cellular entry and massive mitochondrial Ca^2+^ loading (Loftis and Janosky 2003; Stanika et al. 2009). Thus, sustained hypoxia necessarily implies excitotoxicity, worsening in turn the metabolic crisis and death if respiration is not promptly established.

Overstimulation of extrasynaptic NMDA receptors increases nitric oxide (NO) production, and further oxidative stress by formation of peroxynitrite upon its reaction with superoxide anions. NO can directly decrease mitochondrial membrane potentials, liberating pro-apoptotic proteins (Moncada and Bolaños 2006), including AIF, NADPH oxidase, and neuronal nitric oxide synthase (nNOS) (Hwang et al. 2002), provoking DNA fragmentation and mitochondrial fission, maintaining a condition of high ADP/ATP ratio and energy inefficiency (Pérez-Pinzon et al. 1999). Mitochondrial structure, function, and energy metabolism change over time, implicating that the physiology of mitochondria also evolves along the life span of an individual (Mattson 2007).

Upon delivery and during neonatal and early developmental stages, oxidative stress is a permanent risk for the developing individual, enhanced by a sudden increase or decrease of metabolism associated with development itself or environment-dependent conditions, including malnutrition, fatigue, fever, infections, trauma, and/or inflammation-inducing injuries (Deng 2010). Oxidative stress produces an imbalance that favours the production of ROS over antioxidant defences (Orrenius et al. 2011), with hydrogen peroxide (H_2_O_2_) playing a pivotal role (Sies 2017). At low concentrations (1–10 nM), H_2_O_2_ leads to adaptative stress responses, while above 1 μM H_2_O_2_ induces inflammation, growth arrest, and cell death (Deng 2010; Aschbacher et al. 2013).

## An Experimental Model of Global PA in Rats

In our laboratory, we established an experimental model of global PA in rats, originally proposed by Borje Bjelke, Kurt Andersson, and collaborators at the Karolinska Institutet, Stockholm, Sweden, in the 1990s (Bjelke et al. 1991; Andersson et al. 1992; Herrera-Marschitz et al. 1993). In this model, hypoxia occurs at the time when the rats are ready for or have begun delivery. The model has been pivotal for the study of relevant targets responsible for metabolic cascades leading to long-term effects (see Herrera-Marschitz et al. 2011, 2014; recently reviewed by Barkhuizen et al. 2017).

The model starts by a programmed mating. At the time of pro-oestrus, a female Wistar rat is exposed to a male for one night, looking the next day for a vaginal clot to exactly predict the time of delivery (22 days). When on term, a first spontaneous delivery is observed before the dam is neck-dislocated and subjected to hysterectomy to remove the foetus-containing uterine horns, which are immersed in a water bath at 37 °C for 21 min in order to induce severe asphyxia. The foetuses are manually delivered and stimulated to start breathing, and after a nursering period, the pups are given to surrogate dams pending further experiments. Sibling, spontaneous, or caesarean-delivered pups are used as controls (see Herrera-Marschitz et al. 2011). The model allows monitoring early or delayed long-lasting molecular, metabolic, and physiological effects, or the pups can also be used to prepare organotypic cultures (Morales et al. 2003; Klawitter et al. 2007).

PA is a menace to the full organism, affecting systemic and brain tissue. The availability of ATP is rapidly decreased in the kidneys, already after 5 min of PA, whereas brain ATP is decreased to less than 50% after 15 min of asphyxia if performed at 37 °C (Engidawork et al. 1997). Heart metabolism is sustained until the time when the lack of oxygenation is incompatible with life, largely supported by the “phosphocreatine shuttle” (Friedman and Roberts 1994), which is not useful for the neonatal brain (Lubec et al. 2000), although there is some clinical evidence showing that a creatine-supplemented diet protects the newborn from birth hypoxia (Ireland et al. 2008, 2011; Tachikawa et al. 2007), but further research is certainly required to evaluate the phosphocreatine shuttle in the developing brain.

PA implies a primary and a secondary insult. The primary insult relates to the lack of oxygen, and the secondary one to the oxidative stress triggered by re-oxygenation, resulting in the formation of ROS and RNS, and overactivation of glutamate receptors and mitochondrial deficiencies, as recently discussed (Hagberg et al. 2016).

## The Hypoxic Insult

The brain is vulnerable to a decrease of blood oxygen saturation, due to its high dependence on aerobic metabolism. Whenever hypoxia is sustained, there is a switch to glycolysis, a poor metabolic alternative because of the low glucose stores in newborn brain tissue and deficient ATP output by the glycolysis pathway, resulting in lactate accumulation and acidosis (Engidawork et al. 1997). The cerebral energy metabolism of newborn rodents and humans can utilise ketone bodies β-hydroxybutyrate and acetoacetate rather than glucose to satisfy cerebral energy requirements (see Nehlig and Pereira de Vasconcelos, 1993). In neonates, these ketone bodies are essential energy sources, produced by liver mitochondria and diffusing to other organs including the brain. Ketone uptake into the brain of the newborn is four to five times faster than that in older babies or infants (Cunnane and Crawford 2014). In adults, ketones can provide the energy requirements following prolonged fasting or starvation (Wang et al. 2014), and are also the main source of carbon to make cholesterol and long-chain fatty acids, important structural lipids for the developing brain (Cunnane et al. 1999). It is not yet known, however, if ketone bodies can compensate for the energy crisis elicited by hypoxia during the perinatal period.

## The Re-oxygenation Insult

Re-oxygenation is a requirement for survival, leading necessarily to oxidative stress and free radical formation, excitotoxicity, intracellular calcium accumulation, mitochondrial dysfunction, and inactivation of buffering enzymes, resulting in a metabolic deficient condition, increasing CNS vulnerability to recurrent metabolic insults.

Oxidative stress and free radical formation lead to inhibition of Na^+^-dependent glutamate uptake by astroglial cells, the main mechanism regulating extracellular glutamate levels (Herrera-Marschitz et al. 1996; see Anderson and Swanson 2000) implicated in short- and long-term excitoxicity, as discussed by Herrera-Marschitz and Schmidt (2000). The impairment of glial-glutamate transport leads to extracellular glutamate overflow. If not taken up, glutamate binds to extrasynaptic NMDARs, expressing at neonatal stage Ca^2+^-permeable NR2B and Mg^2+^-insensitive NR3A NMDAR subunits (Massey et al. 2004; see Groc et al. 2009; Hardingham and Bading 2010; Jantzie et al. 2015; also, Papouin et al. 2012). Overstimulation of extrasynaptic NMDAR increases Ca^2+^ influx, triggering mitochondrial dysfunction and ROS and RNS formation (Starkov et al. 2004; Stanika et al. 2009), modifying lipids and macromolecules, such as proteins and nucleic acids (see Quincozes-Santos et al. 2014). There is evidence that NMDA receptor blockage improves mitochondrial respiration, preventing mitochondrial permeabilization during the reperfusion phase following hypoxic-ischaemic injury (Block and Schwarz 1996; Chen et al. 1998; Puka-Sundvall et al. 2000).

ROS and RNS levels can overwhelm the capacity of cellular defence systems. ROS introduce post-translational oxidative carbonyl modifications on macromolecules (Lourenco dos Santos et al. 2015), while S-nitrosylation modifies reactive cysteine thiol on target proteins, leading to both protein misfolding and fission/fusion-dependent mitochondrial dysfunction and fragmentation (Nakamura and Lipton 2011). PARP-1 is further activated (Duan et al. 2007; Abramov and Duchen 2008), and AIF is translocated, triggering caspase-independent apoptosis (see Krantic et al. 2007). ROS alter IκB degradation, resulting in NF-κB activation, and nuclear translocation of the p65 subunit, which is increased in a PARP-1-dependent manner by PA (Neira-Peña et al. 2015). The global perinatal insults trigger inflammatory signalling in peripheral and brain tissues, implicating also vascular integrity. Cyclooxygenase-2 (COX-2), a marker of inflammation, is transiently elevated in rat brain exposed to PA, together with up- and subsequent down-regulation of antioxidant enzymes (Toti et al. 2001; Bonestroo et al. 2013). This suggests delayed vulnerability that could contribute to developmental abnormalities responsible for the behavioural alterations observed after PA (Piscopo et al. 2008), also in the ischaemic neonatal human brain (Toti et al. 2001). At neonatal stages, the effect of increased ROS and RNS is aggravated by immature defence mechanisms, including low expression/activity of the superoxide dismutase (SOD) family, glutathione peroxidase (GPx) (Samarasinghe et al. 2000), catalase (Lafemina et al. 2006), and peroxyredoxin-3 (Prx-3) (Chang et al. 2004). Prx-3 is exclusively located in mitochondria, co-localising with SOD-2 (Mn-SOD) (Watabe et al. 1997; Cao et al. 2007). Prx-3 protects against peroxynitrite anions (Hattori et al. 2003; see Hanschmann et al. 2013), and it is expressed heterogeneously, correlating with a regional sensitivity to excitotoxic damage (Hattori et al. 2003; Aon-Bertolino et al. 2011).

## Current Brain-Protecting Strategies

Therapeutic options against the long-term effects of PA are limited and mainly based on hypothermia, which provides protection only if initiated soon after the insult (Thoresen et al. 2013). No consensus on clinical protocols has been achieved yet, and the advantages and disadvantages of head or whole body cooling are still debated, including safety and developmental considerations (Committee on Fetus & Newborn 2014; Allen 2014; Shankaran et al. 2014; Sabir and Cowan 2015; Ahearne et al. 2016).

## Hypothermia

There is compelling clinical evidence that cerebral hypothermia improves the neurodevelopmental outcome when applied to infants with moderate to severe hypoxic-ischaemic encephalopathy (HIE), before the onset of a secondary deterioration phase (Edwards et al. 2010; Guillet et al. 2012; Shankaran et al. 2012; see Wassink et al. 2014; Vanlandingham et al. 2015). This has led to a recommendation supported by the *European Resuscitation Council* that any new therapeutic approach dealing with neonatal encephalopathy should be compared to the effect produced by hypothermia (Davidson et al. 2015; Gunn and Thoresen 2015).

As mentioned above, the current hypothermia protocols are still insufficient (Allen 2014), and a main drawback is the existence of a narrow therapeutic window (Odd et al. 2009; Sabir and Cowan 2015; Ahearne et al. 2016). The rationale of hypothermia is graded reduction of cerebral metabolism, about 5% for every degree Celsius of temperature reduction (Laptook et al. 1995; Erecinska et al. 2003). Cooling also reduces post-depolarization release of excitatory amino acids during hypoxia-ischaemia, both in newborn (Thoresen et al. 1997) and in adult (Nakashima and Todd 1996) subjects. Microdialysis experiments showed that hypothermia initiated immediately after hypoxia-ischaemia in newborn piglets was associated with reduced levels of excitatory amino acids and reduced NO efflux compared to the control condition (Thoresen et al. 1997; Rostami et al. 2013), in agreement with evidence that glutamate antagonists can also prevent events occurring in the early recovery phase following hypoxia-ischaemia, before failure of mitochondrial function takes place, but only when the effect of the antagonist is associated with hypothermia (Nurse and Cobertt 1996).

## PARP-1 Overactivation

PARP-1 plays a critical role during development, and is activated upon any threat to the genome, either for its repair or for initiating cell death signalling to protect its integrity. Indeed, PARP-1 overactivation elicits a NMDAR-dependent, caspase-independent, parthanatos-like cell death programme (Wang et al. 2016) and PA increases PARP-1 activity in the rat brain shortly after the insult (Allende-Castro et al. 2012), triggering a signalling cascade leading to nuclear translocation of the NF-κB subunit p65 and the expression of the pro-inflammatory proteins IL-1β and TNF-α, increasing cell death (Neira-Peña et al. 2015). Such effects are prevented by the PARP-1 inhibitor nicotinamide, supporting previous reports showing similar protection against neuronal death (Klawitter et al. 2007), brain dopaminergic dysfunction (Bustamante et al. 2007), and behavioural deficits (Simola et al. 2008; Morales et al. 2010) assessed 2–6 months after the perinatal insult. PARP-1 inhibition attenuates nNOS activation and reduces cell death induced by oxidative conditions (Pieper et al. 2000; Klawitter et al. 2007), restoring metabolic functions including energy production (Chen et al. 2009; Xu et al. 2009). Nicotinamide, as an NAD^+^ precursor, probably protects against the metabolic cascade elicited by the primary insult, avoiding NAD^+^ exhaustion and the energy crisis.

While the hypothesis of PARP-1 inhibition is promising, it has yet to reach a consensus in order to attempt a clinical trial, in part because nicotinamide does not provide full protection against the effects elicited by PA. Nicotinamide prevents vulnerability to recurrent metabolic insults, but mainly in the substantia nigra, with only minor effects in the neocortex and neostriatum (Neira-Peña et al. 2015; Perez-Lobos et al. 2017). The issue of analogues, precursors, or metabolites of nicotinamide is attractive (Trammell et al. 2016), recently discussed in relation to nicotinamide riboside, which improves mitochondrial and stem cell function, prolonging the life span of mice (Zhang et al. 2016).

Nicotinamide mononucleotide has been shown to be superior to nicotinamide as a precursor of NAD^+^ (Kawamura et al. 2016), leading to the proposal that nicotinamide mononucleotide protects from energy deficits by restoring NAD^+^ and ATP levels, reducing ROS accumulation (Wang et al. 2016). The oral bioavailability of nicotinamide mononucleotide has been reported, and this intermediate mitigates age-associated physiological decline in mice without any obvious toxicity or deleterious effects, enhancing mitochondrial oxidative metabolism and preventing mitonuclear protein imbalance (Mills et al. 2016). It is not yet known whether nicotinamide riboside or mononucleotide can also decrease PARP-1 overactivation, or whether they can prevent the long-term consequences of PA.

## Glutamate Excitotoxicity

The involvement of glutamate in excitotoxicity-mediated damage induced by metabolic insults, including stroke, ischaemia, and hypoxia, led to the strategy of treating with selective high-affinity NMDA and/or AMPA antagonists, all of which failed in clinical trials (Cheng et al. 2004). The classical non-competitive NMDAR antagonist dizocilpine (MK-801) gave hope for death prevention following PA (Herrera-Marschitz et al. 1993, 1994; Engidawork et al. 2001), but it was largely surpassed by hypothermia. It was also discussed whether the minor protection provided by MK-801 is explained by a hypothermia-induced effect (Buchan and Pulsinelli 1990; see Alkan et al. 2001; Makarewicz et al. 2014).

Several glutamate transporter proteins are expressed by astrocytes, mainly GLAST (EAAT1) and GLT-1 (EAAT2) subtypes, responsible for the majority of glutamate uptake (see Robinson and Jackson 2016), providing a target for increasing or decreasing extracellular glutamate levels (Herrera-Marschitz et al. 1996). *N*-Acetylcysteine, a clinically established antioxidant, has been shown to activate the cystine/glutamate antiporter, modulating extracellular glutamate levels (Danbolt 2001; see Berk et al. 2013). The actual direction of the transport of cystine or glutamate depends upon the intracellular and extracellular concentration of the respective molecules. Several clinical studies have shown that *N*-acetylcysteine is well tolerated, promising a role for the treatment of a number of neuropsychiatric disorders (Wink et al. 2016; see Berk et al. 2013). In the brain, *N*-acetylcysteine is deacetylated and oxidised to cystine, which is reduced back to cysteine when taken up by the cells, playing a role in the synthesis of glutathione (GSH) (Bavarsad Shahripour et al. 2014). Thus, *N*-acetylcysteine is perhaps an option to be tested in the present model (see Quintanilla et al. 2016).

## Memantine as a Lead for a Neonatal Protecting Strategy

D145 (1-amino-3,5-dimethyladamantane), better known as memantine, was first proposed as a putative anti-parkinsonian drug in the 1980s, since it induced rotational behaviour in unilaterally 6-OHDA-lesioned animals with a profile mimicking that of d-amphetamine and apomorphine, indirect and direct dopamine agonists respectively (Danysz et al. 1997; see Herrera-Marschitz et al. 2007, 2010). This was confirmed by Seeman et al. (2008), demonstrating the action of memantine on dopamine D_2_ receptors. Nevertheless, the main pharmacodynamic feature supporting a clinical application in stroke, ischaemia, or neurodegenerative disorders was based on the observation that memantine is a low-affinity, use-dependent, NMDAR channel blocker with fast kinetics, not interfering with normal synaptic transmission, but blocking NMDAR only when it is overstimulated (Volbracht et al. 2006; Rammes et al. 2008). Memantine is considered at present as a prototype for targeting extrasynaptic NMDR activity (Garcia-Munoz et al. 2015; Johnson et al. 2015).

Memantine is well tolerated and has a low incidence of adverse effects (Kavirajan 2009), also inducing mild hypothermia (Krieglstein et al. 1997), a feature further supporting its clinical potential (see Rammes et al. 2008). Memantine has been approved by the European Medicine Agency and the Food and Drug Administration (FDA-USA) for the treatment of moderately severe Alzheimer’s disease (2006). It has been reported that memantine can reduce functional and morphological sequelae induced by ischaemia (Block and Schwarz 1996; Chen et al. 1998; Volbracht et al. 2006), possibly by selectively blocking extrasynaptic NMDAR (Chen et al. 1992; Xia et al. 2010; Garcia-Munoz et al. 2015). Memantine has also been evaluated for efficacy in children with pervasive developmental disorders, including leukomalacia, while there is still concern about increasing constitutive apoptosis, recommending further preclinical investigation (Manning et al. 2011).

An important issue refers to whether memantine prevents the excitotoxic cascade elicited by PA. This question merits investigation, either with memantine alone and/or together with the PARP-1 inhibitor nicotinamide, to assess prevention of the short- and long-term effects elicited by PA, profiting also from the mild hypothermia induced by memantine (Krieglstein et al. 1997; Gunn and Thoresen 2015). Memantine can improve mitochondrial respiration, preventing mitochondrial permeabilization during the secondary (reperfusion) phase following hypoxic-ischaemic injury (Block and Schwarz 1996; Chen et al. 1998; Pirinen et al. 2014; Olah et al. 2015), in agreement with a pivotal role of extrasynaptic NMDA receptors in the effects elicited during the re-oxygenation phase following hypoxia.

The chemical structure of memantine (3,5-dimethyltricyclo[3.3.1.1^3,7^]decan-1-amine) allows additional groups to be attached to increase selectivity, improving its pharmacodynamic and/or pharmacokinetic properties. Hence, nitromemantine has been synthesised, by attaching a nitrate group to produce 3-amino-5,7-diethyladamantan-1-yl nitrate. This NO source improved efficacy as a neuroprotectant, compared to that provided by memantine, by nitrosating a redox-mediated regulatory site on the extrasynaptic NMDA receptor (Lipton 2006; Takahashi et al. 2015; see Nakamura and Lipton 2016).

Hypothermia and excitatory amino acid blockage can provide a potent synergism for prevention of the secondary energetic mitochondrial-related failure associated with PA. Nevertheless, many of the compounds used for blocking the initial phases of injury, excitotoxicity, and oxidative stress elicited by hypoxic-ischaemic encephalopathy failed, also because of blockage of normal functions associated to glutamatergic signalling. MK-801 decreases death following PA (Peruche and Krieglstein 1993; Herrera-Marschitz et al. 1993, 1994), but it also induces apoptosis, impairing brain development in rats (Ikonomidou et al. 1999).

## Vulnerability to Recurrent Metabolic Insults

**Fig. 1:**
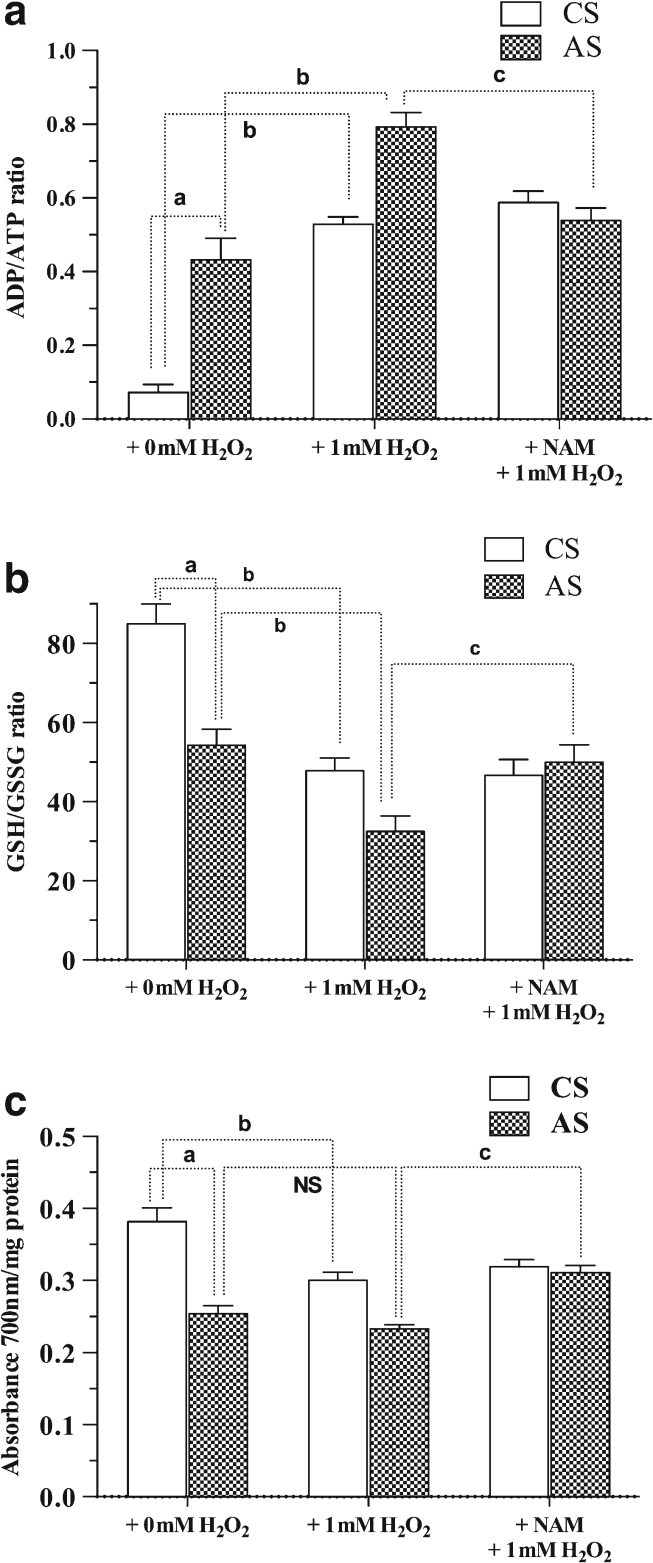
ADP/ATP (**a**), GSH/GSSG (**b**), and )**c**) potassium ferricyanide-reducing power measurements on entire sample homogenates from triple organotypic cultures. **a** ADP/ATP ratio observed in cultures at 21 days in vitro (DIV) from caesarean-delivered (controls; CS) (*open columns*) and asphyxia-exposed (AS) (hatched columns) rat neonates (P2) (means ± SEM; *n* = 6, for each experimental groups). **b** GSH/GSSG ratio. **c** Potassium ferricyanide-reducing power. ^a^ *P* < 0.05 for the effect of asphyxia (CS + Sal versus AS + Sal); ^b^ *P* < 0.05 for the effect of H_2_O_2_ (CS + Sal + 0 mM H_2_O_2_ versus CS + Sal + 1 mM H_2_O_2_, or AS + Sal + 0 mM H_2_O_2_ versus AS + Sal + 1 mM H_2_O_2_). ^c^ *P* < 0.05, for the effect of nicotinamide (NAM) (CS + Sal + 0 mM H_2_O_2_ versus CS + NAM + 0 mM H_2_O_2_; CS + Sal + 1 mM H_2_O_2_ versus CS + NAM + 1 mM H_2_O_2_; AS + Sal + 0 mM H_2_O_2_ versus AS + NAM + 0 mM H_2_O_2_; AS + Sal + 1 mM H_2_O_2_ versus AS + NAM + 1 mM H_2_O_2_) (data from Perez-Lobos et al. 2017)

Neonatal metabolic insults may lead to immediate and/or delayed consequences, with clinical onset at different developmental stages. Understanding the sequence of these events is still sketchy. Nevertheless, it has been discussed that metabolic insults occurring at birth (a first hit) can prime development, increasing the vulnerability to recurrent (secondary and tertiary hit) insults, ultimately challenging postnatal development and maturity. In humans, PA is a risk factor for several psychiatric disorders, including learning deficits and schizophrenia. Conversely, in rodents, PA is associated with delayed cell death, dopamine and histamine transmission deficits and behavioural impairments assessed at adulthood, affecting learning, spatial, and non-spatial memory and anxiety (Simola et al. 2008; Morales et al. 2010; Galeano et al. 2011, 2015; Flores-Balter et al. 2016; Tapia-Bustos et al. 2017). The idea of progressive dysfunction and “first” and successive hit sequences, triggering and perpetuating pathophysiological conditions and/or diseases, has recently been discussed (Marriott et al. 2017; Israel et al. 2017). In agreement with this, it was recently reported that PA implies a long-term energy deficit and oxidative stress, evaluated by the ADP/ATP and GSH/GSSG ratio, respectively, prevented by nicotinamide (Perez-Lobos et al. 2017). Figure 1 shows the effect of PA and a recurrent metabolic insult (1 mM H_2_O_2_) on ADP/ATP (Fig. 1a), GSH/GSSG (Fig. 1b), and potassium ferricyanide-reducing power (Fig. 1c) measurements on entire sample homogenates from triple organotypic cultures from caesarean-delivered and asphyxia-exposed rat neonates, making it evident that PA produces a permanent energy deficit (increased ADP/ATP ratio) and oxidative stress (decreased GSH/GSSG ratio), as well as a permanent deficit in reducing antioxidant power, increasing vulnerability to recurrent insults (Perez-Lobos et al. 2017)

## Conclusions

The present review focuses on the short- and long-term metabolic cascades triggered by PA, identifying pathways that can explain long-term vulnerability and clinical consequences. The Karolinska Institutet model of PA has recently been discussed by a review summarising 25 years of research on global asphyxia in the immature rat brain (Barkhuizen et al. 2017).

PA is still a prominent clinical issue with few therapeutic alternatives preventing its long-term consequences. We have established a unique experimental model of global PA in rats occurring at the time of delivery, identifying relevant targets responsible for metabolic cascades leading to short- and long-term effects, evaluated by in vivo and in vitro experiments (see Herrera-Marschitz et al. 2011, 2014). The model is a referent among those used for studying progressive neurological dysfunction originating early in life (Marriot et al. 2017). PA constitutes a model of neural damage and regeneration for many other conditions such as stroke, where hypoxia/ischaemia is often followed by re-oxygenation and injury.

The discussed model has been compared to that of hypoxia/ischaemia proposed by Rice et al. (1981) (Vannucci and Vannucci 1997; Vannucci et al. 1996, 2005), marshalling the fact that the reviewed model is performed with on-term and not with neonates at P7, the former being premature when compared to the neonatal human brain. The prematurity of the neonatal rat brain has been a subject of controversy, arguing that the degree of maturity depends upon the tissue and the functions selected for comparison (Herrera-Marschitz et al. 2014). Further, the model of Vannucci and collaborators involves ligation of vessels, resulting in a pathophysiology equivalent to focal stroke, not necessarily triggered by global asphyxia (Fellman and Raivio 1997). The here discussed model (i) is a good mimic of human delivery (Romero et al. 2006); (ii) it is largely non-invasive, not involving vessel ligation, ischaemia, or exposure of pulmonary breathing animals to N_2_ inhalation chambers (Fellman and Raivio 1997); (iii) it allows evaluation of short- and long-term consequences of the insult, monitored in the same preparation (Kohlhauser et al. 1999a, b; Simola et al. 2008; Morales et al. 2010); (iv) it is highly reproducible among laboratories (Lubec et al. 1997a, b); and (v) it is considered as a reference by worldwide leading labs (see Wassink et al. 2014; Gunn and Thoresen 2015). Furthermore, (vi) the model is unique in providing a bridge between in vivo and in vitro experiments, based on the same preparation. Indeed, foetuses and neonates can be used to prepare organotypic cultures, complementing and/or allowing experiments with bioethical restrictions when performed in vivo, such as exposing surviving neonates to a recurrent insult (Perez-Lobos et al. 2017).

The organotypic culture model originally developed by BH Gahwiler in Zurich (1981) was validated by Plenz and Kitai (1996a, b; Plenz et al. 1998), as a powerful tool for studying rat basal ganglia neurocircuitries. In this model, the pattern of neuronal innervation and neurocircuitry formation is moved back to an earlier stage, providing an opportunity to monitor under the microscope how neurites and processes look for their corresponding targets, establishing innervation plexuses, showing electrophysiological (Plenz and Kitai 1996b) and neurochemical (Gomez-Urquijo et al. 1999) features similar to those observed in vivo. The model has been used to demonstrate the effect of PA on the number and branching of tyrosine-hydroxylase-positive neurons, illustrating the vulnerability of the dopaminergic systems to PA (Morales et al. 2003; Klawitter et al. 2005, 2007). We have recently reported that the organotypic model allows evaluation of the effect of PA on postnatal vulnerability to oxidative stress, demonstrating an additive effect to that produced by PA on cells with neuronal and glial phenotype, prevented by systemic nicotinamide treatment 1 h after birth (Perez-Lobos et al. 2017).

PA provides a framework to address a fundamental issue affecting long-term CNS plasticity. The perinatal insult triggers a domino-like sequence of events making the developing individual vulnerable to recurrent adverse conditions, decreasing his/her coping repertoire because of a relevant insult occurring at birth (Marriott et al. 2017). The issue of the short- and long-term consequences of PA has heuristic relevance, since PA implicates a long-term biological vulnerability that fully depends on the severity of an insult occurring at birth, independently of any genetic or clinical predisposition (Sahin and Sur 2015; Jain et al. 2017). Indeed, by definition, PA (an environmentally dependent variable) refers to an unexpected interruption of oxygen at the time of delivery, when labour has already begun. Therefore, no genetic factor, malformation, or prematurity is included in the clinical entity of PA, which is defined as a specific metabolic/energetic insult related, first, to the delay and/or interruption of autonomous breathing, and, second, to re-oxygenation, a requirement for survival.

## Abbreviations

ADP: Adenosine diphosphate
AIF: Apoptosis inducing factor
AM: Calcein-acetoxymethyl ester
AMPA: α-Amino-3-hydroxy-5-methyl-4-isoxazolepropionic acid
AS: Asphyxia-exposed rats
ATP: Adenosine triphosphate
BCA: Bicinchoninic acid
Bcl-2: B-cell lymphoma 2
Bnip3: BCL2-interacting protein 3
CNS: Central nervous system
CS: Caesarean-delivered rats
Cx: Neocortex
COX-2: Cyclooxygenase-2
D145: 1-Amino-3,5-dimethyladamantane
DAPI: 4′6-Diamidino-2-phenylindole
DIV: Days in vitro
DNA: Deoxyribonucleic acid
DTNB: 5,5′-Dithiobis-2-nitrobenzoic acid
EthD-1: Ethidium-homodimer-1
FDA: Food and Drug Administration
G: Gestation day
GFAP: Glial fibrillary acidic protein
GLAST (EAAT1): Glutamate aspartate transport
GLT-1 (EAAT2): Glutamate transport-1
GPx: Glutathione peroxidase
GSH: Reduced glutathione
GSSG: Oxidised glutathione
H_2_O_2_: Hydrogen peroxide
HIE: Hypoxic-ischaemic encephalopathy
HIF-1α: Hypoxia-induced factor-1α
i.p.: Intraperitoneal
IL-1β: Interleukin 1β
MAP-2: Microtubule-associated protein-2
MK-801: Dizocilpine
NAC: *N*-Acetylcysteine
NAD^+^: Nicotinamide adenine dinucleotide
NADPH: Nicotinamide adenine dinucleotide phosphate
NAM: Nicotinamide, niacinamide, vitamin B3
NF-κB: Nuclear factor kappa-light-chain-enhancer of activated B cells
Nix: BCL2/adenovirus E1B-interacting protein 3-like
NMDAR: N-Methyl-d-aspartate receptor
nNOS: Neuronal nitric oxide synthase
NO: Nitric oxide
Noxa: Phorbol-12-myristate-13-acetate-induced protein 1
6-OHDA: 6-Hydroxydopamine
ONOO: Peroxynitrite anion
P: Postnatal day
PA: Perinatal asphyxia
PARP-1: Poly(ADP-ribose) polymerase-1
Prx-3: Peroxyredoxin-3
pVHL: von Hippel-Lindau tumour-suppressing factor
RFU: Relative fluorescence units
RNS: Reactive nitrogen species
ROS: Reactive oxygen species
TNF-α: Tumour necrosis factor alpha
TH: Tyrosine hydroxylase
TUNEL: Terminal deoxynucleotidyl transferase dUTP nick end labelling

## References

[CR1] Abramov AY, Duchen MR (2008). Mechanisms underlying the loss of mitochondrial membrane potential in glutamate excitotoxicity. Biochim Biophysis Acta.

[CR2] Ahearne CE, Boylan GB, Murray DM (2016). Short and long term prognosis in perinatal asphyxia: an update. World J Clin Pediatr.

[CR3] Alkan T, Kahveci N, Buyukusal L, Korfali E, Ozluk K (2001). Neuroprotective effect of MK-801 and hypothermia used alone and in combination in hypoxic-ischemic brain injury in neonatal rats. Arch Physiol Biochem.

[CR4] Allen KA (2014). Moderate hypothermia: is selective head cooling or whole body cooling better?. Adv Neonatal Care.

[CR5] Allende-Castro C, Espina-Marchant P, Bustamante D, Rojas-Mancilla E, Neira T, Gutierrez-Hernandez MA, Esmar D, Valdes JL, Morales P, Gebicke-Haerter PJ, Herrera-Marschitz M (2012). Further studies on the hypothesis of PARP-1 inhibition as strategy for lessening the long-term effects produced by perinatal asphyxia: effects of nicotinamide and theophylline on PARP-1 activity in brain and peripheral tissue. Neurotox Res.

[CR6] Anderson CM, Swanson RA (2000). Astrocyte glutamate transport: review of properties, regulation and physiological functions. Glia.

[CR7] Andersson K, Bjelke B, Bolme P, Ögren S-Ö (1992). Asphyxia-induced lesion of the rat hippocampus (CA1, CA3) and the nigro-striatal dopamine system. In: Gross J (ed) Hypoxia and ischemia. CNS. Wissenschafliche Publikationen der Humboldt-Universitat zu Berlin, B. Medizin.

[CR8] Aon-Bertolino ML, Romero JI, Galeano P, Holubec M, Badorry MS, Saraceno GE, Hanschmann E-M, Lillig CH, Capani F (2011). Thioredoxin and glutaredoxin system protein-immunocolocalization in the rat central nervous system. Biochemica et Biophysica Acta.

[CR9] Aschbacher K, O'Donovan A, Wolkowitz OM, Dhabhar FS, Su Y, Epel E (2013). Good stress, bad stress and oxidative stress: insights from anticipatory cortisol reactivity. Psychoneuroendocrinology.

[CR10] Barkhuizen M, van den Hove DLA, Vles JSH, Steinbusch HWM, Kramer BW, Gavilanes AWD (2017). 25 years of research on global asphyxia in the immature rat brain. Neurosci Biobehav Revs.

[CR11] Basovich SN (2010). The role of hypoxia in mental development and in the treatment of mental disorders: a review. Biosci Trends.

[CR12] Bavarsad Shahripour R, Harigan MR, Alexndrov AV (2014). N-cetylcysteine (NAC) in neurological disorders: mechanisms of action and therapeutic opportunities. Brain Behaviour.

[CR13] Berger NA (1985). Poly (ADP-ribose) in the cellular response to DNA damage. Radiat Res.

[CR14] Berk M, Malhi GS, Gray LJ, Dean OM (2013). The promise of N-acetylcistein in neuropsychiatry. Trends Pharmacol Sci.

[CR15] Bjelke B, Andersson K, Ogren SO, Bolme P (1991). Asphyctic lesion: proliferation of tyrosine hydroxylase immunoreactive nerve cell bodies in the rat substantia nigra and functional changes in dopamine neurotransmission. Brain Res.

[CR16] Block F, Schwarz M (1996). Memantine reduces functional and morphological consequences induced by global ischemia in rats. Neurosci Lett.

[CR17] Bonestroo HJ, Nijboer CH, van Velthoven CT, Kavelaars A, Hack CE, van Bel F, Heinjnen CJ (2013). Cerebral and hepatic inflammatory response after neonatal hypoxia-ischemia in newborn rats. Dev Neurosci.

[CR18] Bruick RK (2000). Expression of the gene encoding the proapoptotic Nip3 protein is induced by hypoxia. Proc Natl Acad Sci U S A.

[CR19] Buchan A, Pulsinelli WA (1990). Hypothermia but not the N-methyl-D-aspartate antagonist MK-801, attenuates neuronal damage in gerbils subjected to transient global ischemia. J Neurosci.

[CR20] Bustamante D, Morales P, Torres-Pereyra J, Goiny M, Herrera-Marschitz M (2007). Nicotinamide prevents the effect of perinatal asphyxia on dopamine release evaluated with in vivo microdialysis 3 months after birth. Exp Brain Res.

[CR21] Cao Z, Lindsay JG, Isaacs NW (2007). Mitochondrial peroxiredoxins. Subcell Biochem.

[CR22] Chang TS, Cho CS, Park S, Yu S, Kang SW, Rhee SG (2004). Peroxiredoxin III, a mitochondrion-specific peroxidase, regulates apoptotic signaling by mitochondria. J Biol Chem.

[CR23] Chen H-SV, Pellegrini JW, Aggarwal SK, Lei SZ, Warach S (1992). Open-channel block of N-methyl-D-aspartat (NMDA) responses by memantine: therapeutic advantage against NMDA-mediated neurotoxicity. J Neurosci.

[CR24] Chen HS, Wang YF, Rayudu PV, Edgecomb P, Neill JC, Segal MM, Lipton SA, Jensen FE (1998). Neuroprotective concentrations of the N-methyl-D-aspartate open channel blocker memantine are effective without cytoplasmic vacuolation following post-ischemic administration and do not block maze learning or long-term potentiation. Neuroscience.

[CR25] Chen L, Liu L, Yin J, Luo Y, Huang S (2009). Hydrogen peroxide-induced neuronal apoptosis is associated with inhibition of protein phosphatase 2-A and 5, leading to activation of MAPK pathway. Int J Biochem Cell Biol.

[CR26] Cheng YD, Al-Khoury L, Zivin JA (2004). Neuroprotection for ischemic stroke: two decades of success and failure. NeurotoxRx.

[CR27] Committee on Fetus & Newborn (Papile LA, Baley JE, Benitz W, Cummings J, Carlo WA, Eichenwald E, Kumar P, Pollin RA, Tan RC, Wang KS) (2014) Hypothermia and neonatal encephalopathy. Am Acad Pediatr, 1098–1275; doi: 10.1542/peds.%202014-0899 10.1542/peds.2014-0899 24864176

[CR28] Cunnane SC, Menard CR, Likhodii SS, Brenna JT, Crawford MA (1999). Carbon recycling into de novo lipogenesis is a major pathway in neonatal metabolism of linoleate and alpha-linolenate. Prostsg Leukotr Ess.

[CR29] Cunnane SC, Crawford MA (2014). Energetic and nutritional constraints on infant brain development: implications for brain expansion during human evolution. J Human Evol.

[CR30] Danbolt NC (2001). Glutamate uptake. Prog Neurobiol.

[CR31] Danysz W, Parsons CG, Kornhuber J, Schmidt WJ, Quack G (1997). Aminoadamantanes as NMDA receptor antagonists and antiparkinsonian agents—preclinical studies. Neurosci Biobehav Rev.

[CR32] Davidson JO, Green CR, Bennet L, Gunn AJ (2015) Battle of the hemichannels-Connexins and Pannexins in ischemic brain injury. Int J Develop Neurosci. doi: 10.1016/EP%202014.12.07 10.1016/j.ijdevneu.2014.12.007 25546019

[CR33] Deng W (2010). Neurobiology of injury to the developing brain. Nat Rev Neurol.

[CR34] Douglas-Escobar M, Weiss MD (2015). Hypoxic-ischemic encephalopathy; a review for the clinician. JAMA Pediatr.

[CR35] Duan Y, Gross RA, Sheu SS (2007). Ca2+-dependent generation of mitochondrial reactive oxygene species serves as a signal for poly(ADP-ribose) polymerase-1 activation during glutamate excitotoxicity. J Physiol.

[CR36] Edwards AD, Brockhurst P, Gunn AJ, Halliday H, Juszczak E, Levene M, Strohm B, Thoresen M, Withelaw A, Azzopardi D (2010) Neurological outcomes at 18 months of age after moderate hypothermia for perinatal hypoxic ischaemic encephalophaty: synthesis and meta-analysis of trial data. Brit Med J. doi: 10.1136/bmj.c363 10.1136/bmj.c363 PMC2819259 20144981

[CR37] Engidawork E, Chen Y, Dell’Anna E, Goiny M, Lubec G, Ungerstedt U, Andersson K, Herrera-Marschitz M (1997). Effects of perinatal asphyxia on systemic and intracerebral glycolysis metabolism and pH in the rat. Exp Neurol.

[CR38] Engidawork E, Loidl F, Chen Y, Kohlhauser C, Stoeckler S, Dell’Anna E, Lubec B, Lubec G, Goiny M, Gross J, Andersson K, Herrera-Marschitz M (2001). Comparison between hypothermia and glutamate antagonism treatments on the immediate outcome of perinatal asphyxia. Exp Brain Res.

[CR39] Erecinska M, Thoresen M, Silver IA (2003). Effects of hypothermia on energy metabolism in: mammalian central nervous system. J Cereb Blood Flow Metab.

[CR40] Fellman V, Raivio KO (1997). Reperfusion injury as the mechanism of brain damage after perinatal asphyxia. Pediatr Res.

[CR41] Flores-Balter G, Cordova-Jadue H, Chiti-Morales A, Lespay C, Espina-Marchant P, Falcon R, Grinspun N, Sanchez J, Bustamante D, Morales P, Herrera-Marschitz M, Valdés JL (2016). Effect of perinatal asphyxia on tuberomammillary nucleus neuronal density and object recognition memory: a possible role for histamine?. Behav Brain Res.

[CR42] Friedman DL, Roberts R (1994). Compartmentation of brain-type creatine kinase and ubiquitous mitochondrial creatine kinase in neurons: evidence for a creatine phosphate energy shuttle in adult brain. J Comp Neurol.

[CR43] Galeano P, Blanco-Calvo E, Madureira-de Oliveira D, Cuenya L, Kamenetzky GV, Mustaca AE, Barreto GE, Giraldez-Alvarez LD, Milei J, Capani F (2011). Long-lasting effects of perinatal asphyxia on exploration, memory and incentive downshift. Int J Dev Neurosci.

[CR44] Galeano P, Blanco E, Logica Tornatore TM, Romero JI, Holubiec MI, Rodríguez de Fonseca F, Capani F (2015). Life-long environmental enrichment counteracts spatial learning, reference and working memory deficits in middle-aged rats subjected to perinatal asphyxia. Front Behav Neurosci.

[CR45] Garcia-Munoz M, Lopez-Huerta V, Carrillo-Reid L, Arbuthnott G (2015). Extrasynaptic glutamate NMDA receptors: key players in striatal function. Neuropharmacology.

[CR46] Gahwiler BH (1981). Organotypic monolayer cultures of the nervous tissue. J Neurosci Methods.

[CR47] Glasow NC, Retchless B, Johnson JW (2015). Molecular bases of NMDA receptor subtype-dependent properties. J Physiol.

[CR48] Gomez-Urquijo S, Hokfelt T, Ubink R, Lubec G, Herrera-Marschitz M (1999). Neurocircuitries of the basal ganglia studied in organotypic cultures: focus on tyrosine hydroxylase, nitric oxide synthase and neuropetide immunocytochemistry. Neuroscience.

[CR49] Gonzalez-Flores A, Aguilar-Quesada R, Siles E, Pozo S, Rodriguez-Lara MI, Lopez-Jimenez L, Lopez-Rodriguez M, Peralta-Leal A, Villar D, Martin-Oliva D del Peso L, Berra E, Oliver FJ (2014) Interaction between PARP-1 and HIF-2alpha in the hypoxic response. Oncogene 33:891–898. 10.1038/onc.2013.9 23455322

[CR50] Groc L, Bard L, Choquet D (2009). Surface trafficking of N-methyl-D-aspartate receptors: physiological and pathological perspectives. Neuroscience.

[CR51] Gunn AJ, Thoresen M (2015) Animal studies of neonatal hypothermic neuroprotection have translated well in to practice. J Resuscitation. doi: 10.1016/j.resuscitation.2015.03.026 10.1016/j.resuscitation.2015.03.026 25930163

[CR52] Hagberg H, Edwards AD, Groenendaal F (2016). Perinatal brain damage: the term infant. Neurobiol Dis.

[CR53] Hanschmann E-M, Godoy JR, Berndt C, Hudemann C, Lillig CH (2013). Thioredoxins, glutaterodoxins, and peroxiredoxins—molecular mechanisms and health. Significance: from cofactors to antioxidants to redox signalling. Antioxid Redox Signal.

[CR54] Hardingham GE, Bading H (2010). Synaptic versus extrasynaptic NMDA receptor signalling: implications for neurodegenerative disorders. Nat Rev Neurosci.

[CR55] Hattori F, Murayama N, Noshita T, Oikawa S (2003). Mitochondrial peroxiredoxin-3 protects hippocampal neurons from excitototxic injury in vivo. J Neurochem.

[CR56] Herrera-Marschitz M, Loidl CF, Andersson K, Ungerstedt U (1993). Prevention of mortality induced by perinatal asphyxia: hypothermia or glutamate anstagonism?. Amino Acids.

[CR57] Herrera-Marschitz M, Loidl CF, You Z-B, Andersson K, Silveira R, O’Connor WT, Goiny M (1994). Neurocircuitry of the basal ganglia studied by monitoring neurotransmitter release. Effects of intracerebral and perinatal asphyctic lesions. Mol Neurobiol.

[CR58] Herrera-Marschitz M, Goiny M, Meana JJ, Silveira R, Godukhin O, Chen Y, Espinoza S, Pettersson E, Loidl F, Lubec G, Andersson K, Nylander I, Terenius L, Ungerstedt U (1996). On the origin of extracellular glutamate levels monitored in the basal ganglia by in vivo microdialysis. J Neurochem.

[CR59] Herrera-Marschitz M, Schmidt WJ (2000). Amino acids in neurobiology (eds). Amino Acids.

[CR60] Herrera-Marschitz M, Bustamante D, Morales P, Goiny M (2007). Exploring neurocircuitries of the basal ganglia by intracerebral administration of selective neurotoxins. Neurotox Res.

[CR61] Herrera-Marschitz M, Arbuthnott G, Ungerstedt U (2010). The rotational model and microdialysis: significance for dopamine signalling, clinical studies and beyond. Prog Neurobiol.

[CR62] Herrera-Marschitz M, Morales P, Leyton L, Bustamante D, Klawitter V, Espina-Marchant P, Allende C, Lisboa F, Cunich G, Jara-Cavieres A, Neira T, Gutierrez-Hernandez MA, Gonzalez-Lira V, Simola N, Schmitt A, Morelli M, Andrew Tasker R, Gebicke-Haerter PJ (2011). Perinatal asphyxia: current status and approaches towards neuroprotective strategies, with focus on sentinel proteins. Neurotox Res.

[CR63] Herrera-Marschitz M, Neira-Pena T, Rojas-Mancilla E, Espina-Marchant P, Esmar D, Perez R, Munoz V, Gutierrez-Hernandez MA, Rivera B, Simola N, Bustamante D, Morales P, Gebicke-Haerter PJ (2014). Perinatal asphyxia: CNS development and deficits with delayed onset. Front Neurosci.

[CR64] Hong SJ, Dawson TM, Dawson VL (2004). Nuclear and mitochondrial conversations in cell death: PARP-1 and AIF signalling. Trends in Pharmacol Sci.

[CR65] Hwang J-J, Choi S-Y, Koh J-Y (2002). The role of NADPH oxidase, neuronal nitric oxide synthase and poly(ADP ribose) polymerase in oxidative neuronal death induced in cortical cultures by brain-derived neurotrophic factor and neurotrophin-4/5. J Neurochem.

[CR66] Ikonomidou C, Bosch F, Miksa M, Bittigau P, Vockler J, Dikranian K, Tenkova TI, Stefovska V, Turski L, Olney JW (1999). Blockade of NMDA receptors and apoptotic neurodegeneration in the developing brain. Science.

[CR67] Ireland Z, Dickinson H, Snow R, Walker DW (2008). Maternal creatine: does it reach the fetus and improve survival after an acute hypoxic episode in the spiny mouse (Acomys caharinus)?. Am J Obstet Gynecol.

[CR68] Ireland Z, Castillo-Melendez M, Dickison H, Snow R, Walker DW (2011). A maternal diet supplemented with creatine from mid-pregnancy protecs the nwborn spiny mousee brain from birth hypoxia. Neuroscience.

[CR69] Israel Y, Karahanian E, Ezquer F, Morales P, Ezquer M, Rivera-Meza M, Herrera-Marschitz M, Quintanilla ME (2017). Acquisition, maintenance and relapse-like alcohol drinking: lessons from the UChB rat line. Front Behav Neurosci.

[CR70] Jacobucci GJ, Popescu GK (2017). NMDA receptors: linking physiological output to biophysical operation. Nature Rev.

[CR71] Jain SV, Mathur A, Srinivasakumar P, Wallendorf M, Culver JP, Zempel JM (2017). Prediction of neonatal seizures in hypoxic-ischemic encephalopathy using electroencephalograph power analyses. Pediatr Neurol.

[CR72] Jantzie LL, Talos DM, Jackson MC, Park H-K, Graham DA, Lechpammer M, Folkern RD, Volpe JJ, Jensen FE (2015). Developmental expression of N-methyl-D-aspartate (NMDA) receptor subunits in human white and gray matter: potential mechanism of increased vulnerability in the immature brain. Cereb Cortex.

[CR73] Johnson JW, Glasgow NG, Polysheva NV (2015). Recent insights into the mode of action of memantine and ketamine. Curr Opinion Pharmacol.

[CR74] Kavirajan H (2009). Memantine a comprensive review of safety and efficacy. Expert Opinion Drug Saf.

[CR75] Kawamura T, Mori N, Shibata K (2016). β-Nicotinamide mononucleotide, an anti-aging cadidate compoun, is retained in the body for longer than nicotinamide in ratas. J Nutr Sci Vitaminol (Tokyo).

[CR76] Ke Q, Costa M (2006). Hypoxia-inducible factor-1 (HIF-1). Mol Pharmacol.

[CR77] Klawitter V, Morales P, Johansson S, Bustamante D, Goiny M, Gross J, Luthman J, Herrera-Marschitz M (2005). Effect of perinatal asphyxia on cell survival, neuronal phenotype and neurite growth evaluated with organotypic triple cultures. Amino Acids.

[CR78] Klawitter V, Morales P, Bustamante D, Gomez-Urquijo S, Hökfelt T, Herrera-Marschitz M (2007). Plasticity of basal ganglia neurocircuitries following perinatal asphyxia: neuroprotection by nicotinamide. Exp Brain Res.

[CR79] Kohlhauser C, Mosgoeller W, Hoeger H, Lubec G, Lubec B (1999). Cholinergic, monoaminergic and glutamatergic changes following perinatal asphyxia in the rat. Cell Mol Life Sci.

[CR80] Kohlhauser C, Kaehler S, Mosgoeller W, Singewald N, Koulevas D, Prast H, Hoeger H, Lubec B (1999). Histological changes and neurotransmitter levels three months following perinatal asphyxia in the rat. Life Sci.

[CR81] Krantic S, Mechawar N, Reix S, Quirion R (2007). Apoptosis–inducing factor: a matter of neuron life and death. Prog Neurobiol.

[CR82] Krieglstein J, Seif el Nasr M, Lipper K (1997). Neuroprotection by memantine as increased by hypothermia and nimodipine. Eur J Pharm Sci.

[CR83] Kurinczuk JJ, White-Konig M, Badawi N (2010). Epidemiology of neonatal encephalopathy and hypoxic-ischemic encephalopathy. Early Hum Dev.

[CR84] Lafemina MJ, Sheldon RA, Ferreiro DM (2006). Acute hypoxia-ischemia results in hydrogen proxide accumulation in neonatal but not adult mouse brain. Pediatric Res.

[CR85] Laptook AR, Cirbett RJ, Sterett R, Garcia D, Tollfsbol G (1995). Quantitative relationship between brain temperature and energy utilization measured in vivo using 31P and 1H magnetic resonance spectroscopy. Pediatr Res.

[CR86] Lipton SA (2006). Paradigm shift in neuroprotection by NMDA receptor blockade: memantine and beyond. Nat Rev Drug Discov.

[CR87] Loftis JM, Janosky A (2003). The N-methyl-D-aspartate receptor subunit NR2B: localization, functional properties, regulation and clinical implications. Pharmacol Ther.

[CR88] Lourenco dos Santos S, Baraibar MA, Lundberg S, Eeg-Olofsson O, Larsson L, Friguet B (2015). Oxidative proteome alterations during skeletal muscle aging. Redox Biol.

[CR89] Low JA (2004). Determining the contribution of asphyxia to brain damage in the neonate. J Obstet Gynaecol Res.

[CR90] Lubec B, Marx M, Herrera-Marschitz M, Labudova O, Hoeger H, Gille L, Nohl H, Mosgoeller W, Lubec G (1997). Decrease of heart protein kinase C and cyclin-dependent kinase precedes death in perinatal asphyxia of the rat. FASEB J.

[CR91] Lubec B, Dell’Anna E, Fang-Kircher S, Marx M, Herrera-Marschitz M, Lubec G (1997). Decrease of brain protein kinase C, protein kinase A, and cyclin-dependent kinase correlating with pH precedes neuronal death in neonatal asphyxia. J Investig Med.

[CR92] Lubec B, Chiappe-Gutierrez M, Hoeger H, Kitzmueller E, Lubec G (2000). Glucose transporters, hexokinases and phosphofructokinase in brain of rats with perinatal asphyxia. Pediatric Res.

[CR93] Makarewicz D, Sulejczak D, Duszczyk M, Malek M, Slomka M, Lazarewicz JW (2014). Delayed preconditioning with NMDA receptor antagonist in a rat model of perinatal asphyxia. Folia Neuropathol.

[CR94] Manning SM, Griffin B, Fitzgerald E, Selip DB, Volpe JJ, Jensen FE (2011). The clinically available NMDA receptor antagonist, memantine, exhibits relative safety in the developing rat brain. Int J Dev Neurosci.

[CR95] Marriott AL, Rojas-Mancilla E, Morales P, Herrera-Marschitz M, Tasker RA (2017). Models of progressive neurological dysfunction originating early in life. Prog Neurobiol.

[CR96] Martin-Oliva D, Aguilar-Quezada R, Ovalle F, Muñoz-Gamez JA, Martinez-Romero R, García del Moral R, Ruiz de Almodovar JM, Villuendas R, Piris MA, Oliver FJ (2006). Inhibition of poly(ADP-ribose) polymerase modulates tumor-related gene expression, including hypoxia inducible factor-1 activation during skin carcinogenesis. Cancer Res.

[CR97] Martinez-Romero R, Canuelo A, Martinez-Lara E, Oliver J, Cardenas S, Siles E (2009). Poly(ADP-ribose) polymerase-1 modulation of in vivo response of brain hypoxia-inducible factor-1 to hypoxia/reoxygenation is mediated by nitric oxide and factor inhibiting HIF. J Neurochem.

[CR98] Massey PV, Johnson BE, Moult PR, Auberson YP, Brown MW, Molnar E, Collingridge GL, Bashir ZI (2004). Differential roles of NR2A and NR2B-containing NMDA receptors in cortical long-tem potentiation and long-term depression. J Neurosci.

[CR99] Mattson M (2007). Mitochondrial regulation of neuronal plasticity. Neurochem Res.

[CR100] Mills KF, Yoshida S, Stein LR, Grozio A, Kubota S, Sasaki Y, Redpath P, Migaud ME, Apte RS, Uchid K, Yoshino J, Imai SI (2016). Long-term dministration of nicotinamide mononucleotide mitigates age-associated physiological decline in mice. Cell Metab.

[CR101] Moncada S, Bolaños JP (2006). Nitric oxide, cell bioenergetics and neurodegeneration. J Neurochem.

[CR102] Morales P, Klawitter V, Johansson S, Huaiquín P, Barros VG, Avalos AM, Fiedler J, Bustamante D, Gomez-Urquijo S, Goiny M, Herrera-Marschitz M (2003). Perinatal asphyxia impairs connectivity and dopamine neurite branching in organotypic triple culture from rat substantia nigra, neostriatum and neocortex. Neurosci Lett.

[CR103] Morales P, Fiedler JL, Andres S, Berrios C, Huaiquin P, Bustamante D, Cardenas S, Parra E, Herrera-Marschitz M (2008). Plasticity of hippocampus following perinatal asphyxia: effects on postnatal apoptosis and neurogenesis. J Neurosci Res.

[CR104] Morales P, Simola N, Bustamante D, Lisboa F, Fiedler J, Gebicke-Haerter P, Morelli M, Tasker RA, Herrera-Marschitz M (2010). Nicotinamide prevents the long-term effect of perinatal asphyxia on apoptosis, non-spatial working memory and anxiety in rats. Exp Brain Res.

[CR105] Nakamura T, Lipton SA (2011). Redox modulation by S-nitrosylation contributes to protein misfolding, mitochondria dynamics and neuronal synaptic damage in neurodegenerative diseases. Cell Death Differ.

[CR106] Nakamura T, Lipton SA (2016). Protein S-nitrosylation as a therapeutic target for neurodegenerative diseases. Trends Pharmacol Sci.

[CR107] Nakashima K, Todd MM (1996). Effects of hypothermia on the rate of excitatory amino acid release after ischemic depolarization. Stroke.

[CR108] Neira-Peña T, Rojas-Mancilla E, Munoz-Vio V, Perez R, Gutierrez-Hernandez M, Bustamante D, Morales P, Hermoso MA, Gebicke-Haerter P, Herrera-Marschitz M (2015). Perinatal asphyxia leads to PARP-1 overactivity, p65 translocation, IL-1β and TNF-α overexpression, and apoptotic-like cell death in mesencephalon of neonatal rats: prevention by systemic neonatal nicotinamide administration. Neurotox Res.

[CR109] Nehlig A, Pereira de Vasconcelos A (1993). Glucose and ketone body utilization by the brain neonatal rat. Prog Neurobiol.

[CR110] Nurse S, Cobertt D (1996). Neuroprotection after several days of mild, drug-induced hypothermia. J Cereb Blood Flow Metab.

[CR111] Odd DE, Lewis G, Whitelaw A, Gunnell D (2009). Resuscitation at birth and cognition at 8 years of age: a cohort study. Lancet.

[CR112] Olah G, Szczesny B, Brunyanski A, Lopez-Garcia IA, Gero D, Radak Z, Szabo C (2015) Differentiation-associated down regulation of ply(ADP-ribose) polymerase-1 expression in myoblasts serves to increase their resistance to oxidative stress. PLoS One 10(7). 10.1371/journal.pne.0134227 10.1371/journal.pone.0134227 PMC4517814 26218895

[CR113] Orrenius S, Nicotera P, Zhivotovsky B (2011). Cell death mechanisms and their implications in toxicology. Toxicol Sci.

[CR114] Pan R, Chen C, Liu WL, Liu KJ (2013). Zinc promotes the death of hypoxic astrocytes by upregulating hypoxia-induced hypoxia-inducible factor-1alpha expression via poly(ADP-ribose) polymerase-1. CNS Neurosci Ther.

[CR115] Papouin T, Ladepeche L, Ruel J, Sacchi S, Labasque M, Hanini M, Groc L, Pollegioni L, Mothet J-P, Oliet SHR (2012). Synaptic and extrasynaptic NMDA receptors are gated by different endogenous coagonists. Cell.

[CR116] Perez-Lobos R, Lespay-Rebolledo C, Tapia-Bustos A, Palacios E, Vio V, Bustamante D, Morales P, Herrera-Marschitz M (2017) Vulnerability to a metabolic challenge following perinatal asphyxia evaluated by organotypic cultures: neonatal nicotinamide treatment. Neurotox Res. doi: 10.1007/s12640-017-9755-4 10.1007/s12640-017-9755-4 28631256

[CR117] Pérez-Pinzón MA, Xu GP, Born J, Lorenzo J, Busto R, Rosenthal M, Sick TJ (1999). Cytochrome C is released from mitochondria into the cytosol after cerebral anoxia or ischemia. J Cereb Blood Flow Metab.

[CR118] Peruche B, Krieglstein J (1993). Mechanisms of drug actions against neuronal damage caused by ischemia—an overview. Prog Neuro-Psychopharmacol Biol Psychiat.

[CR119] Petralia RS (2012) Distribution of extrasynaptic NMDA receptors on neurons. Sci World J. doi: 10.1100/2012/267120 10.1100/2012/267120 PMC3361219 22654580

[CR120] Pieper AA, Walles T, Wei G, Clements EE, Verma A, Snyder SH, Zweier JL (2000). Myocardial postischemic injury is reduced by poly-ADPribose polymerase-1 gene disruption. Mol Med.

[CR121] Pirinen E, Canto C, Jo YS, Morato L, Zhang H, Menzies KJ, Williams EG, Mouchiroud L, Moullan N, Hagberg C, Li W, Timmers S, Imhof R, Verbeek J, Pujol A, van Loon B, Viscomi C, Zeviani M, Schrauwen P, Sauve AA, Schoonjans K, Auwrrk J (2014). Pharmacological inhibition of poly(ADP-ribose) polymerases improves fitness and mitochondrial function in skeletal muscle. Cell Metab.

[CR122] Piscopo P, Bernardo A, Calamandrei G, Venerosi A, Valanzano BD, Confaloni A, Minghetti L (2008). Altered expression of cyclooxygenase-2, presenilins and oxygen radical scavenging enzymes in a rat model of global perinatal asphyxia. Exp Neurol.

[CR123] Plenz D, Kitai ST (1996). Organotypic cortex-striatum-mesencephalon cultures: the nigrostriatal pathway. Neurosci Lett.

[CR124] Plenz D, Kitai ST (1996). Generation of high frequency oscillations in cortical circuits of somatosensory cortex cultures. J Neurophysiol.

[CR125] Plenz D, Herrera-Marschitz M, Kitai ST (1998). Morphological organization of the subthalamic nucleus-globus pallidus system studied in organotypic cultures. J Comp Neurol.

[CR126] Puka-Sundvall M, Hallin U, Zhu C, Wang X, Karlsson JO, Blomgren K, Hagberg H (2000). NMDA blockade attenuates caspase-3 activation and DNA fragmentation after neonatal hypoxia-ischemia. Neuroreport.

[CR127] Quincozes-Santos A, Bobermin LD, Tramontina AC, Wartchow KM, Taglisri B, Souza DA, Wyse ATS, Goncalves C-A (2014) Oxidtive stress mediated by NMDA, AMPA/KA channels in acute hippocampal slices: neuroprotective effect of resveratrol. Toxicol in Vitro 28: 544–551. 10.1016/j.tiv.2013.12.021 24412540

[CR128] Quintanilla ME, Rivera-Meza M, Berríos-Cárcamo P, Salinas-Luypaert C, Herrera-Marschitz M, Israel Y (2016). Beyond the “first hit”: marked inhibition by N-acetyl cysteine of chronic ethanol intake but not of early ethanol intake. Parallel effects on ethanol-induced saccharin motivation. Alcohol Clin Exp Res.

[CR129] Rammes G, Danysz W, Parsons CG (2008). Pharmacodynamics of memantine: an update. Curr Neuropharmacol.

[CR130] Rice JE, Vannucci RC, Briley JB (1981). The influence of immaturity on the hypoxic-ischemic brain damage in the rat. Ann Neurol.

[CR131] Robinson MB, Jackson JG (2016). Astroglial glutamate transporter coordinate excitatory signaling and brain energetics. Neurochem Int.

[CR132] Romero R, Espinoza J, Kusanovic JP, Gotsch F, Hassan S, Erez O, Chaiworapongsa T, Mazor M (2006) The preterm parturition syndrome. BJOG (Suppl 3): 17-42. 10.1111/j.1471-0528.2006.01120.x PMC7062298 17206962

[CR133] Romero R, Dey SK, Fisher SJ (2014). Preterm labor: one syndrome, many causes. Science.

[CR134] Rostami E, Rockesen D, Ekberg NR, Goiny M, Ungerstedt U (2013). Brain metabolism and oxygenation in healthy pigs receiving hypoventilation and hyperoxia. Respir Physiol Neurobiol.

[CR135] Sabir H, Cowan FN (2015). Prediction of outcome methods assessing short- and long-term outcomes after therapeutic hypothermia. Sem Fetal Neonatal Med.

[CR136] Sahin M, Sur M (2015) Genes, circuits, and precision therapies for autism and related neurodevelopmental disorders. Science 350(6263). doi: 10.1126/science.aab3897 10.1126/science.aab3897 PMC4739545 26472761

[CR137] Samarasinghe DA, Tapner M, Farrel GC (2000). Role of oxidative stress in hypoxia-reoxygenation injury to cultured rt hepatic sinusoidal endothelial cells. Hepatology.

[CR138] Seeman P, Caruso C, Lasaga M (2008). Memantine agonist action at dopamine D2 high receptors. Synapse.

[CR139] Shankaran S, Pappas A, McDonald SA (2012). Childhood outcomes after hypothermia for neonatal encephalopathy. N Engl J Med.

[CR140] Shankaran S, Laptook AR, Pappas A, McDonald SA, Tyson JE, Poindexter BB, Schibler K, Bell EF, Heyne RJ, Pedroza C, Bara R, Van Meurs KP, Grisby C, Huitema CM, Garg M, Ehrenkranz RA, Shepherd EG, Chalak LF, Hamrick SE, Khan AM, Reynolds AM, Laughon MM, Truog WE, Sysart KC, Carlo WA, Walsh MC, Watterberg KL, Higging RD (2014). Effect of depth and duration of cooling on deaths in the NICU among neonates with hypoxic ischemic encephalopathy: a randomized clinical trial. JAMA.

[CR141] Sies H (2017). Hydrogen peroxide as a central redox signalling molecule in physiological oxidative stress: oxidative eustress. Redox Biol.

[CR142] Simola N, Bustmante D, Pinna D, Pontis S, Morales P, Morelli M, Herrera-Marschitz M (2008). Acute perinatal asphyxia impairs non-spatial memory and alters motor coordination in adult male rats. Exp Brain Res.

[CR143] Sowter HM, Ratcliffe PJ, Watson P, Greenberg AH, Harris AL (2001). HIF-1-dependent regulation of hypoxic induction of the cell death factors BNIP3 and NIX in human tumors. Cancer Res.

[CR144] Stanika RL, Pivovarova NB, Brantner CA, Watts CA, Winters CA, Andrews SB (2009). Coupling diverse routes of calcium entry to mitochondrial dysfunction and glutamate excitotoxicity. PNAS.

[CR145] Starkov AA, Chinopopulos C, Fiskum G (2004). Mitochondrial calcium and oxidative stress as mediators of ischemic brain injury. Cell Calcium.

[CR146] Tachikawa M, Hosoya K, Ohtsuki S, Terasaki T (2007). A novel relationship between creatine transport at the blood-brain and blood retinal barriers, creatine biosynthesis, and its use for brain and retinal homeostasis. Subcell Biochem.

[CR147] Takahashi H, Xia CJ, Talantova M, Bodhinathan K, Li W, Holland EA, Tong G, Pina-Crespo J, Zhang D, Nakanishi N, Larrick JW, McKercher SR, Nakamura T, Wang Y, Lipton SA (2015). Pharmacologically targeted NMDA receptor antagonism by nitromemantine for cerebrovascular disease. Sci Rep.

[CR148] Tapia-Bustos A, Lobos-Perez R, Vio V, Lespay-Rebolledo C, Palacios E, Chiti-Morales A, Bustamante D, Herrera-Marschitz M, Morales P (2017). Moduylation of postnatal neurogenesis by perinatal asphyxia: effect of D1 and D2 dopamine receptor agonists. Neurotox Res.

[CR149] Thoresen M, Satas S, Puka-Sundvall M, Whitelaw A, Hallström A, Loberg EM, Ungerstedt U, Steen PA, Hagberg H (1997). Post-hypoxic hypothermia reduces cerebrocortical release of NO and excitotoxins. Neuroreport.

[CR150] Thoresen M, Tooley J, Liu X, Jary S, Fleming P, Luyt K, Jain A, Cairns P, Harding D, Sabir H (2013). Time is brain: starting therapeutic hypothermia within three hours after birth improves motor outcome in asphyxiated newborns. Neonatology.

[CR151] Toti P, Felice DE, Schürfeld K, Stumpo M, Bartolommei S, Lombardi A, Petraglia E, Buonocore G (2001). Cyclooxygenase-2 immunoreactivity in the ischemic neonatal human brain. An autopsy study. J Submicro Cytol Pathol.

[CR152] Trammell SA, Schmidt MS, Wedemann BJ, Redpath P, Jaksch F, Dellinger RW, Li Z, Abel ED, Migaud ME, Brenner C (2016). Nicotinamide riboside is uniquely and orally bioavailable in mice and humans. Nat Commun.

[CR153] Trotti D, Lodi-Rizzini B, Rossi D, Haugeto O, Racagni G, Danbolt N, Volterra A (1997). Neuronal and glial glutamate transporters possess an SH-based redox regulatory mechanism. EJN.

[CR154] Vangeison G, Carr D, Federoff HJ, Rempe DA (2008). The good, the bad, and the cell type-specific roles of hypoxia inducible factor-1 alpha in neurons and astrocytes. J Neurosci.

[CR155] Vanlandingham SC, Kurz MC, Wang HE (2015). Thermodynamic aspects of therapeutic hypothermia. Resuscitation.

[CR156] Vannucci RC, Vannucci SJ (1997). A model of perinatal hypoxic-ischemic brain damage. Ann N Y Acad Sci.

[CR157] Vannucci RC, Brucklacher RM, Vannucci SJ (1996). The effect of hyperglycemia on cerebral metabolism during hypoxia-ischemia in the immature rat. J Cer Blood Flow Metab.

[CR158] Vannucci RC, Brucklacher RM, Vannucci SJ (2005). Glycolysis in perinatal hypoxic-ischemic brain damage. Dev Neurosci.

[CR159] Vizi ES, Kisfali M, Lorincz T (2013). Role of nonsynaptic GluN2B-containing NMDA receptors in excitotoxicity: evidence that fluoxetine selective inhibits these receptors and may have neuroprotective effects. Brain Res Bull.

[CR160] Volbracht C, van Beek J, Zhu C, Blomgren K, Leist M (2006). Neuroprotective properties of memantine in different in vitro and in vivo models of excitotoxicity. Eur J Neurosci.

[CR161] Wang GL, Jiang BH, Rue EA, Semenza GL (1995). Hypoxia-inducible factor 1 is a basic-helix-loop-helix-PAS heterodimer regulated by cellular O2 tension. Proc Natl Acad Sci U S A.

[CR162] Wang SP, Yang H, Wu JW, Gauthier N, Fukao T, Mitchell GA (2014). Metabolism as a tool for understanding human brain evolution: lipid energy metabolism as an example. J Hum Evol.

[CR163] Wang SN, Xu TY, Li WL, Miao CY (2016) Targetting nicotinamide phosphoribosyltransferase as a potential therapeutic strategy to restore adult neurogenesis. CNS Neurosci Ther 22: 431–439 10.1111/cns.12539 PMC6492912 27018006

[CR164] Wang Y, Xu TY, Li WL, Miao CY (2016). Targeting nicotinamide phosphoribosyltransferase as a potential therapeutic strategy to restore adult neurogenesis. Science.

[CR165] Wassink G, Gunn ER, Drury PR, Bennet L, Gunn AJ (2014) The mechanisms and treatment of asphyxia encephalopathy. Frontiers Neurosci 8:40. doi: 10.3389/fnins.2014.00040.eCollection2014 10.3389/fnins.2014.00040 PMC3936504 24578682

[CR166] Watabe S, Hiroi T, Yamamoto Y, Fujioka Y, Hasegawa H, Yago N, Takahashi Y (1997). SP-22 is a thioredoxin-dependent peroxide reductase in mitochondria. Eur J Biochem.

[CR167] Wink LK, Adams R, Wang Z, Klauning JE, Plawecki MH, Posey DJ, McDougle CJ, Erickson CA (2016). A randomized placebo-controlled pilot study of N-acetylcysteine in youth with autism spectrum disorder. Mol Autism.

[CR168] Xia P, Chen HS, Zhang D, Lipton SA (2010). Memantine preferentially blocks extrasynaptic over synaptic NMDA receptors. J Neurosci.

[CR169] Xu L, Voloboueva LA, Ouyang Y, Emery J, Giffard R (2009). Overexpression of mitochondrial Hsp70/Hsp75 in rat brain protects mitochondria, reduces oxidative stress, and protects from focal ischemia. J Cereb Blood Flow Metab.

[CR170] Zhang H, Ryu D, Wu Y, Gariani K, Wang X, Luan P, D’Amico D, Ropelle ER, Lutolf MP, Aebersold R, Schoonlas K, Menzies KJ, Auwerx J (2016). NAD+ repletion improves mitochondrial and stem cell function and enhances life span in mice. Science.

